# A Reusable SERS Substrate with Internal Standard for the Detection of N-Butylamine Gas

**DOI:** 10.3390/ma19061207

**Published:** 2026-03-19

**Authors:** Mingyang Xu, Xin Li, Lin Xie, Qin Wang, Gang Shi

**Affiliations:** 1 Key Laboratory of Synthetic and Biotechnology Colloids, Ministry of Education, School of Chemical and Material Engineering, Jiangnan University, Wuxi 214122, China; 2 Center of Special Cosmetic Testing (Jiangsu Province), Wuxi Institute of Inspection, Testing and Certification, Wuxi 214101, China

**Keywords:** surface-enhanced Raman scattering, internal standard, photocatalysis, reusability, gas sensing

## Abstract

Surface-enhanced Raman scattering (SERS) has become an effective and sensitive analysis tool for the detection of various molecules. Nevertheless, it is a challenge to fabricate reusable SERS substrates for detecting gaseous molecules. Here, a self-calibrated and reusable SERS substrate has been developed for the quantitative analysis of n-butylamine. The obtained substrate enhances gas enrichment capability through the coordination interaction of Fe_2_O_3_ with the porous structure of ZIF-8, and strengthens the Raman signal intensity by the localized surface plasmon resonance of Ag nanoparticles. Ethanethiol is employed as an internal standard to enhance analysis accuracy. The substrate exhibits excellent quantitative analysis (linear correlation coefficient, R^2^ = 0.996), signal uniformity (RSD = 6.3%), and batch reproducibility (RSD = 4.8%). Moreover, the substrate achieves self-cleaning through photocatalysis. After five cycles, the substrate retains high SERS activity (RSD = 3.13%), exhibiting excellent reusability.

## 1. Introduction

n-Butylamine (BA) is an important organic amine that is widely used in food processing, medical diagnostics, and pharmaceuticals [1,2,3]. However, BA poses significant risks to human health and environmental safety due to its toxicity, volatility, flammability, and corrosiveness [4,5]. Therefore, the rapid and accurate detection of BA is of great importance. To date, various analytical methods have been employed for the quantitative analysis of BA, such as chromatography [6], chemiresistive method [7,8], colorimetry [9,10], and fluorescence spectroscopy [11]. However, these methods often suffer from low sensitivity, and are long time-consuming, which limit their practical applications.

Surface-enhanced Raman scattering (SERS) has garnered significant attention in recent years for its high sensitivity, excellent selectivity, and rapid response [12,13,14]. At present, various SERS substrates have been developed for detecting amine gases. Plasmonic metal nanoparticles (PMNPs) on the substrates leverage the localized surface plasmon resonance (LSPR) effect to generate abundant hotspots, which significantly enhances the Raman signal to improve the detection sensitivity for gas molecules [12,15,16,17]. Owing to the low concentration, weak affinity and rapid diffusion of gas molecules, it remains challenging for PMNP substrates to effectively capture and immobilize gas molecules within hotspot regions [18,19]. To address these challenges, PMNPs have been integrated with porous metal–organic frameworks (MOFs) to fabricate MOF-based composite SERS substrates [14,20,21,22]. MOFs possess large specific surface areas and abundant active sites, which enhance the interaction between gas molecules and the substrate. In addition, their porous structure slows down gas diffusion and captures gas, and then facilitates gas enrichment within hotspots, which realizes the ultrasensitive gas detection [23].

However, these SERS substrates based on noble metal materials cannot be reused after a single use, significantly increasing detection cost and limiting practical application of SERS technology [24,25]. Therefore, developing reusable SERS substrates for BA detection is crucial. Here, a novel SERS substrate with self-calibration and reusability was developed for detecting BA. Fe_2_O_3_ captures BA through the coordination interaction, and ZIF-8 enhances the BA capture efficiency through the porous structure. Moreover, ethanethiol linked to Ag NPs by Ag-S bonds is employed as an internal standard to enhance detection performance. In addition, BA can be precisely removed through photocatalysis to realize the reuse of SERS substrates. This work provides a new strategy for developing reusable SERS substrates for gas detection and exhibits great potential for practical applications.

## 2. Materials and Methods

### 2.1. Materials

n-Butylamine (BA), tetraethyl orthosilicate (TEOS), ethanol, ferric chloride (FeCl_3_), ammonia water (25%), silver nitrate (AgNO_3_), methanol, polyvinylpyrrolidone (PVP), 2-methylimidazole (2-MeIm), and ethanethiol (ET) were purchased from Sinopharm Chemical Reagent Co., Ltd. (Shanghai, China). Urea and 4-aminothiophenol (4-ATP), zinc nitrate hexahydrate (Zn(NO_3_)_2_·6H_2_O) were purchased from Shanghai Aladdin Biochemical Technology Co., Ltd. (Shanghai, China). Si wafers (100) were purchased from Grinm Advanced Materials Co., Ltd. (Nanjing, China).

### 2.2. Synthesis of SiO_2_ [26]

TEOS (3.5 mL), ethanol (92 mL), ammonia water (2.5 mL), and water (17.5 mL) were sequentially added to a flask and stirred at 400 rpm for 6 h. The samples were separated by centrifugation and washed alternately with water and ethanol to obtain SiO_2_ microspheres.

### 2.3. Synthesis of SiO_2_@Fe_2_O_3_ [27]

SiO_2_ (0.1 g) was dispersed in water (40 mL), and then the dispersion was transferred to a flask. Urea (30 mg) and FeCl_3_ (50 mg) were added to the flask. Then, the flask was placed in an oil bath at 95 °C and stirred at 600 rpm for 8 h. After cooling, the samples were separated by centrifugation and washed alternately with water and ethanol to obtain SiO_2_@Fe_2_O_3_.

### 2.4. Synthesis of SiO_2_@Fe_2_O_3_-Ag [28]

SiO_2_@Fe_2_O_3_ (0.1 g), methanol (10 mL) and AgNO_3_ solution (10 mL, 0.02 M) were mixed in water (10 mL). The mixture was transferred to a photoreactor and purged with N_2_ for 30 min to displace oxygen. The photoreactor was placed under UV light of 365 nm for 60 min. The samples were separated by centrifugation and washed alternately with water and ethanol to obtain SiO_2_@Fe_2_O_3_-Ag.

### 2.5. Synthesis of SiO_2_@Fe_2_O_3_-Ag@ZIF-8 [29]

SiO_2_@Fe_2_O_3_-Ag (0.1 g) and PVP (0.1 g) were mixed in methanol (20 mL). After stirring for 30 min, 2-MeIm solution (10 mL, 0.05 M) was added. After continuing stirring for 10 min, Zn(NO_3_)_2_·6H_2_O solution (10 mL, 0.2 M) was added, and the mixture was stirred at room temperature for 10 h. The samples were separated by centrifugation and washed alternately with ethanol to obtain SiO_2_@Fe_2_O_3_-Ag@ZIF-8.

### 2.6. Synthesis of Si/SiO_2_@Fe_2_O_3_-Ag(ET)@ZIF-8 Substrate

SiO_2_@Fe_2_O_3_-Ag@ZIF-8 (10 mg) and ET (5 μL) were mixed in ethanol (10 mL). After stirring for 10 h, the samples were separated by centrifugation and washed with ethanol to obtain SiO_2_@Fe_2_O_3_-Ag(ET)@ZIF-8. Then, the product (10 mg) was dispersed in ethanol (10 mL) and then spin-coated (4000 rpm, 20 s) on Si wafer. The substrate was vacuum-dried to obtain Si/SiO_2_@Fe_2_O_3_-Ag(ET)@ZIF-8 substrate.

### 2.7. Characterization

The surface morphology and elemental composition of the samples were characterized by scanning electron microscopy (SEM, HITACHI S-4800, HITACHI Ltd., Tokyo, Japan) and energy dispersive X-ray spectroscopy (EDS, OCTANE SUPER, AMETEK, Inc., Berwyn, PA, USA). The ultraviolet-visible (UV-vis) absorption spectra were collected by UV-vis spectrophotometer (PERSEE TU-1950, Beijing Purkinje General Instrument Co., Ltd., Beijing, China). The characteristic functional groups of the samples were analyzed by Fourier-transform infrared spectroscopy (FT-IR, Nicolet 6700, Thermo Fisher Scientific, Waltham, MA, USA). The crystal structure of the samples was investigated by X-ray diffractometer (XRD, Bruker AXS D8, Bruker AXS GmbH, Karlsruhe, Germany).

### 2.8. SERS Measurements

The Si/SiO_2_@Fe_2_O_3_-Ag(ET)@ZIF-8 substrate was placed in a closed detection device, and different concentrations of BA solution (5 μL) were added to the device. The device was then placed on an 80 °C hotplate to generate BA gas. After adsorption for 6 h, the Raman spectra were collected on Si/SiO_2_@Fe_2_O_3_-Ag(ET)@ZIF-8 substrate by micro confocal Raman spectrometer (inVia, Renishaw Trading Ltd., London, UK) Measurement parameters are as follows: excitation wavelength is 532 nm, power is 0.05 mW, integration time is 1 s, and number of acquisitions is 1. The detection device is a cylindrical glass container with a volume of 665.37 mL and is equipped with a fan to enhance gas diffusion. The distance between the BA source and the substrate is 4.5 cm. The gas concentration (C_g_, ppb) is calculated according to the following equation [30]: C_g_ = (C_s_ × Q × M × V_m_)/V × 10^9^. Here, C_s_ is the solution concentration (M), Q is the solution volume (L), M is the molar mass of the substance (g·mol^−1^), V_m_ is the molar volume of gas (22.4 L·mol^−1^), and V is the volume of the device (L).

## 3. Results and Discussion

### 3.1. Fabrication and Characterization of SiO_2_@Fe_2_O_3_-Ag@ZIF-8

**Figure 1:**
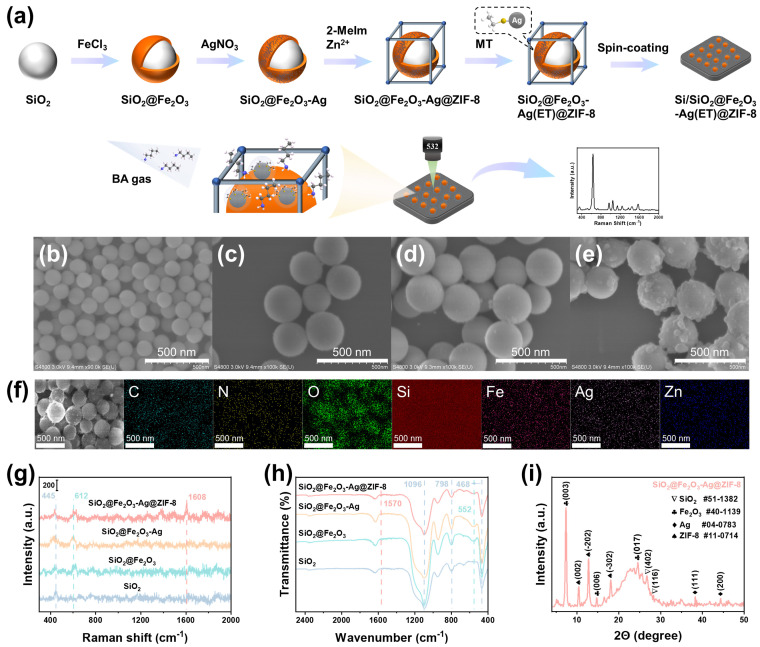
(**a**) Schematic illustration of Si/SiO_2_@Fe_2_O_3_-Ag(ET)@ZIF-8 fabrication and quantitative detection of BA. SEM images of (**b**) SiO_2_, (**c**) SiO_2_@Fe_2_O_3_, (**d**) SiO_2_@Fe_2_O_3_-Ag, and (**e**) SiO_2_@Fe_2_O_3_-Ag@ZIF-8. (**f**) EDS mapping images of SiO_2_@Fe_2_O_3_-Ag@ZIF-8. (**g**) Raman spectra and (**h**) FT-IR spectra of SiO_2_, SiO_2_@Fe_2_O_3_, SiO_2_@Fe_2_O_3_-Ag, and SiO_2_@Fe_2_O_3_-Ag@ZIF-8. (**i**) XRD pattern of SiO_2_@Fe_2_O_3_-Ag@ZIF-8.

The fabrication process of SiO_2_@Fe_2_O_3_-Ag@ZIF-8 is illustrated in Figure 1a. First, SiO_2_ microspheres were synthesized by the Stöber method [26,31]. Figure 1b shows the smooth SiO_2_ microspheres with a diameter of ~200 nm. Subsequently, Fe_2_O_3_ was loaded onto the surface of the SiO_2_ microspheres by the solution-synthesis method to obtain SiO_2_@Fe_2_O_3_ [27]. Figure 1c shows the rough SiO_2_@Fe_2_O_3_ microspheres with a diameter of ~250 nm. To endow the composite with SERS activity, Ag NPs were then deposited onto the SiO_2_@Fe_2_O_3_ by a photochemical reduction method (Figure 1d) [28]. Finally, to enhance enrichment capability toward gas molecules, ZIF-8 was grown on the SiO_2_@Fe_2_O_3_-Ag to obtain SiO_2_@Fe_2_O_3_-Ag@ZIF-8 [29]. As shown in Figure 1e, ZIF-8 forms a continuous coating on the SiO_2_@Fe_2_O_3_-Ag. Figure 1f shows the EDS elemental mapping image of SiO_2_@Fe_2_O_3_-Ag@ZIF-8. The elements O, Si, Fe, and Ag are concentrated in the central region of the microspheres, while the characteristic elements C, N, and Zn of ZIF-8 are distributed on the surface of the microspheres. The above results verify the successful formation of the SiO_2_@Fe_2_O_3_-Ag@ZIF-8. To further investigate the composition of SiO_2_@Fe_2_O_3_-Ag@ZIF-8, the Raman spectra of SiO_2_, SiO_2_@Fe_2_O_3_, SiO_2_@Fe_2_O_3_-Ag and SiO_2_@Fe_2_O_3_-Ag@ZIF-8 were collected (Figure 1g). In the spectrum of SiO_2_, the peak at 445 cm^−1^ is attributed to Si-O-Si stretching vibration [32]. In the spectrum of SiO_2_@Fe_2_O_3_, the new peak at 612 cm^−1^ is assigned to Fe_2_O_3_ [33,34]. In the spectrum of SiO_2_@Fe_2_O_3_-Ag@ZIF-8, besides the above observed peaks, an additional peak at 1608 cm^−1^ is attributed to methyl bending mode of ZIF-8 [35,36]. These findings indicate the successful fabrication of SiO_2_@Fe_2_O_3_-Ag@ZIF-8. The composition of the samples was also analyzed by FI-IR spectra (Figure 1h). The characteristic absorption bands at 468 cm^−1^, 798 cm^−1^, and 1096 cm^−1^ correspond to the O-Si-O bending vibration, symmetric stretching vibration and asymmetric stretching vibration of SiO_2_, respectively [30,37]. The absorption band at 552 cm^−1^ is attributed to the Fe-O stretching vibration of Fe_2_O_3_ [27,38]. The absorption band observed at 1570 cm^−1^ is attributed to the C=N stretching vibrations of ZIF-8 [39]. These findings also verify the successful formation of SiO_2_@Fe_2_O_3_-Ag@ZIF-8. Figure 1i shows the XRD pattern of SiO_2_@Fe_2_O_3_-Ag@ZIF-8. The diffraction peaks of SiO_2_, Fe_2_O_3_, Ag, and ZIF-8 simultaneously appeared in the pattern [34,40], which further confirms the successful fabrication of SiO_2_@Fe_2_O_3_-Ag@ZIF-8.

### 3.2. Sensing Performance of Si/SiO_2_@Fe_2_O_3_-Ag(ET)@ZIF-8

**Figure 2:**
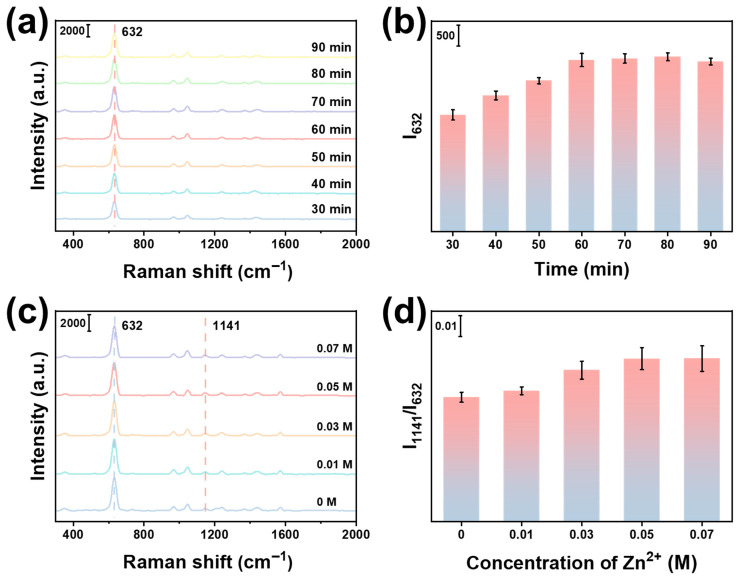
(**a**) Raman spectra and (**b**) I_632_ of Si/SiO_2_@Fe_2_O_3_-Ag(ET) with different illumination time. (**c**) Raman spectra and (**d**) I_1141_/I_632_ of Si/SiO_2_@Fe_2_O_3_(BA)-Ag(ET)@ZIF-8 with different concentrations of Zn^2+^.

To obtain the strongest Raman signal, the Ag NP loading of the SERS substrate was optimized by adjusting the illumination time during photochemical deposition. The Raman spectra were collected from the Si/SiO_2_@Fe_2_O_3_-Ag adsorbed with ET (Si/SiO_2_@Fe_2_O_3_-Ag(ET)). As shown in Figure 2a,b, the intensity of the characteristic Raman peak of ET at 632 cm^−1^ (I_632_) first increases and then decreases with increasing illumination time. When the illumination time is 60 min, the value of I_632_ reaches maximum. This is because a too-short illumination time will lead to insufficient loading of Ag NPs, resulting in less ET adsorption and reduced hotspot density [41]. In contrast, too long a time will lead to excessive deposition and agglomeration of Ag NPs, which weakens the Raman enhancement and hinders the adsorption of ET molecules on the surface of Fe_2_O_3_, thus decreasing the Raman signal intensity of ET [42]. In addition, the porous structure of ZIF-8 plays a crucial role in the enrichment of gas molecules [18]. To achieve optimal enrichment capacity of BA molecules, the loading of ZIF-8 was optimized by changing the concentration of Zn^2+^ during synthesis. The Raman spectra were collected from the Si/SiO_2_@Fe_2_O_3_-Ag(ET)@ZIF-8 adsorbed with BA (Si/SiO_2_@Fe_2_O_3_(BA)-Ag(ET)@ZIF-8). As shown in Figure 2c,d, the ratio of peak intensity at 1141 cm^−1^ (I_1141_) to I_632_ (I_1141_/I_632_) gradually increases with increasing Zn^2+^ concentration. When the concentration of Zn^2+^ is 0.05 M, I_1141_/I_632_ reaches maximum, indicating that the loading of ZIF-8 is optimal under this condition. The substrate adsorbs a large number of BA molecules in the hotspot region by the gas enrichment effect of ZIF-8, thus exhibiting the maximum I_1141_/I_632_.

**Figure 3:**
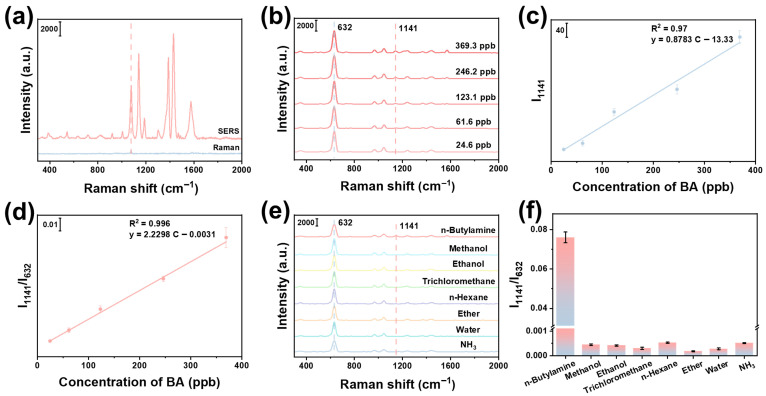
(**a**) Raman spectra of 1 M 4-ATP on the blank Si wafer and 10^−4^ M 4-ATP on the Si/SiO_2_@Fe_2_O_3_-Ag@ZIF-8 substrate. (**b**) Raman spectra of BA with different concentrations. Linear relationship between (**c**) I_1141_ and (**d**) I_1141_/I_632_ and the BA concentration. (**e**) Raman spectra and (**f**) I_1141_/I_632_ of Si/SiO_2_@Fe_2_O_3_-Ag@ZIF-8 adsorbed with different gaseous molecules.

The Raman signal amplification of the substrate was evaluated by the enhancement factor (EF). The EF calculation equation is as follows [43]:

(1) $$EF=\frac{I_{SERS}\times N_{Raman}}{I_{Raman}\times N_{SERS}}$$

where *I_SERS_* and *N_SERS_* are the Raman signal intensity and the number of molecules on the SERS substrate, respectively. *I_Raman_* and *N_Raman_* are the Raman signal intensity and the number of molecules on the blank substrate, respectively. Here, 10 μL of 1 M 4-ATP and 10 μL of 10^−4^ M 4-ATP were added onto a blank Si wafer and the Si/SiO_2_@Fe_2_O_3_-Ag@ZIF-8 substrate, respectively. As shown in Figure 3a, the Raman peak at 1078 cm^−1^ was selected for EF calculation. The values of *I_SERS_* and *I_Raman_* are 7422.6 and 506.9, respectively. Based on the calculation, the EF of the Si/SiO_2_@Fe_2_O_3_-Ag@ZIF-8 substrate is 1.46 × 10^5^.

Quantitative analysis is essential for evaluating SERS substrates [44]. To evaluate the quantitative analysis performance of the developed substrate, the Raman spectra were collected from the Si/SiO_2_@Fe_2_O_3_-Ag(ET)@ZIF-8 adsorbed with different concentrations of BA, namely Si/SiO_2_@Fe_2_O_3_(BA)-Ag(ET)@ZIF-8. As shown in Figure 3b, I_1141_ increases gradually with increasing BA concentration, while I_632_ remains basically unchanged. Figure 3c shows a linear relationship between I_1141_ and BA concentration (y = 0.8783 C − 13.33, R^2^ = 0.97). To further improve detection reliability, the internal standard method was employed to correct signal fluctuations. There is a good linear relationship between I_1141_/I_632_ and BA concentration (y = 2.2298 C − 0.0031, R^2^ = 0.996), as shown in Figure 3d. According to the equation LOD = 3.3σ/S [45], where σ represents the standard deviation value of the blank sample response (n = 10), and S represents the slope of the calibration curve, the limit of detection (LOD) is 1.6 ppb. The Fe_2_O_3_ in the substrate has unsaturated Fe binding sites, while BA has -NH_2_ groups, and BA can be adsorbed on the surface of Fe_2_O_3_ by coordination interaction [46]. In addition, the porous structure of ZIF-8 slows BA gas diffusion and facilitates the entry of gas molecules into the SERS hotspot area [18]. Therefore, the substrate achieves synergistic enhancement of gas enrichment capability, resulting in a low detection limit. These results indicate that the developed substrate has excellent quantitative detection capability for BA, and the sensitivity and accuracy of the developed substrate are effectively improved by the internal standard method. Common gaseous molecules (methanol, ethanol, trichloromethane, n-hexane, ether, water, NH_3_) were employed as interferents to evaluate the selectivity of the developed substrate for BA. As shown in Figure 3e,f, the developed substrate has no response to the above interferents at 1141 cm^−1^, which is attributed to the absence of C-N stretching vibration of these interferents and the lack of strong coordination with Fe_2_O_3_ [47]. Therefore, the substrate exhibits excellent selectivity for BA. In addition, the performance of the Si/SiO_2_@Fe_2_O_3_-Ag(ET)@ZIF-8 substrate was compared with that of other reported SERS substrate in the field of BA detection. As summarized in Table 1, the developed substrate developed in this study demonstrates superior quantitative analysis capability for BA.

**Figure 4:**
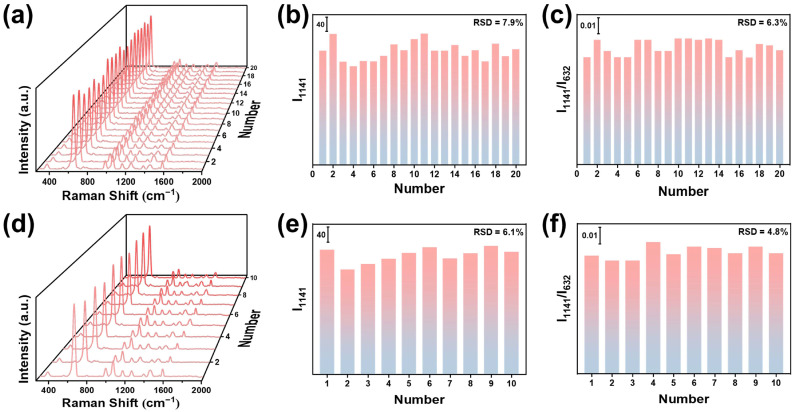
(**a**) Raman spectra of 20 random spots on the same SiO_2_@Fe_2_O_3_(BA)-Ag(ET)@ZIF-8. (**b**) I_1141_ and (**c**) I_1141_/I_632_ derived from (**a**). (**d**) Raman spectra of 10 different batches of SiO_2_@Fe_2_O_3_(BA)-Ag(ET)@ZIF-8. (**e**) I_1141_ and (**f**) I_1141_/I_632_ derived from (**d**).

Signal uniformity and batch reproducibility are also crucial for evaluating the performance of SERS substrates. To evaluate signal uniformity, the Raman spectra were collected from 20 randomly selected spots on the same Si/SiO_2_@Fe_2_O_3_(BA)-Ag(ET)@ZIF-8 under the same conditions (Figure 4a). As shown in Figure 4b, the relative standard deviation (RSD) calculated based on I_1141_ is 7.9%. After the correction through the internal standard method, the signal fluctuations caused by the measurement errors are weakened. The RSD based on I_1141_/I_632_ decreases to 6.3% (Figure 4c), indicating that the SERS substrate has excellent signal uniformity. To evaluate batch reproducibility, 10 batches of Si/SiO_2_@Fe_2_O_3_(BA)-Ag(ET)@ZIF-8 substrates were fabricated under the same method, and the Raman spectra were collected under the same conditions (Figure 4d). As shown in Figure 4e, the RSD calculated based on I_1141_ is 6.1%. After correction through the internal standard method, the RSD based on the I_1141_/I_632_ decreases to 4.8% (Figure 4f). The results indicate that the SERS substrate has excellent batch reproducibility.

### 3.3. Photocatalytic Self-Cleaning Capability of Si/SiO_2_@Fe_2_O_3_-Ag(ET)@ZIF-8

**Figure 5:**
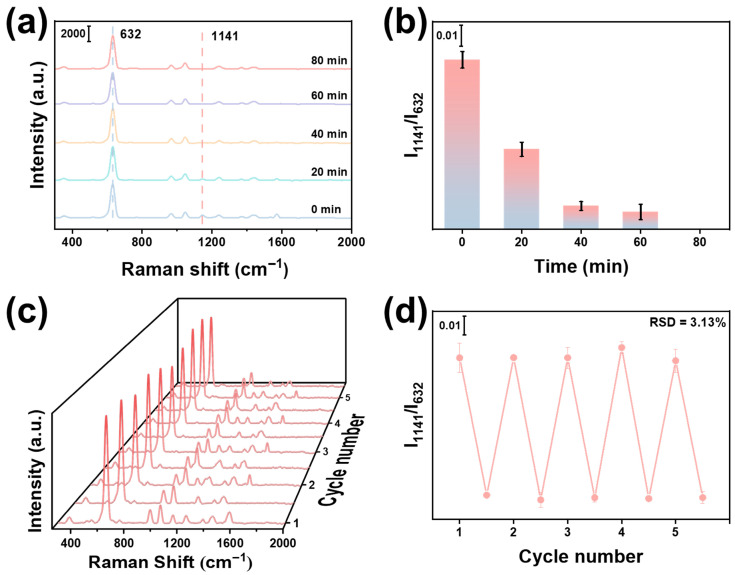
(**a**) Raman spectra and (**b**) I_1141_/I_632_ of Si/SiO_2_@Fe_2_O_3_(BA)-Ag(ET)@ZIF-8 with different illumination times. (**c**) Raman spectra and (**d**) I_1141_/I_632_ of Si/SiO_2_@Fe_2_O_3_(BA)-Ag(ET)@ZIF-8 during 5 adsorption–degradation cycles.

To investigate the optimal UV illumination time for photocatalytic self-cleaning, the Si/SiO_2_@Fe_2_O_3_(BA)-Ag(ET)@ZIF-8 substrate was immersed in water and exposed to UV light (wavelength 365 nm, power density 5 mW·cm^−2^). The Raman spectra were collected after different illumination times. As shown in Figure 5a, with increasing illumination time, the characteristic Raman peak of BA at 1141 cm^−1^ gradually decreases, while the peak at 632 cm^−1^ remains basically unchanged. When the illumination time is 80 min, I_1141_/I_632_ completely disappears (Figure 5b), indicating that the BA molecules adsorbed on Fe_2_O_3_ have been completely degraded. Therefore, the optimal illumination time for photocatalytic self-cleaning is 80 min. To assess the reusability of the SERS substrate, the Si/SiO_2_@Fe_2_O_3_(BA)-Ag(ET)@ZIF-8 was immersed in water and exposed to UV light for 80 min. Subsequently, the Raman spectra before and after UV exposure were collected. The adsorption and degradation processes were repeated 5 times, accompanied by the appearance and disappearance of the characteristic peaks of BA for 5 times (Figure 5c). Figure 5d shows the variation of I_1141_/I_632_ over 5 cycles. Notably, the substrate retains high SERS activity after 5 cycles, and the RSD is 3.13%. These results indicate that the Si/SiO_2_@Fe_2_O_3_-Ag(ET)@ZIF-8 exhibits excellent photocatalytic activity due to the heterostructure of Fe_2_O_3_ and Ag. The adsorbed BA can be degraded by photocatalysis to achieve outstanding self-cleaning capability, resulting in its reusability.

## 4. Conclusions

In conclusion, a self-calibrated and reusable SERS substrate was successfully developed for quantitative detection of BA gas. The substrate consists of SiO_2_@Fe_2_O_3_, Ag NPs and ZIF-8. Fe_2_O_3_ captures BA through the coordination interaction, and the porous ZIF-8 enhances gas capture efficiency. In addition, ET is employed as an internal standard to correct signal fluctuations and improve detection accuracy. The substrate exhibited excellent quantitative analysis (LOD = 1.6 ppb, R^2^ = 0.996), signal uniformity (RSD = 6.3%), and batch reproducibility (RSD = 4.8%). More importantly, the reusability of the substrate can be achieved through photocatalysis, and the high SERS activity after 5 cycles (RSD = 3.13%) still remains. This study designs a reusable SERS substrate for accurate detection of amine gases with promising application prospects.

## Figures and Tables

**Table 1:** Comparison of BA detection methods.

Analytical Method	R^2^	LOD	Reference
SERS sensor	0.996	1.6 ppb	This work
Fluorescence sensor	0.971	47.2 ppm	[48]
Fluorescence sensor	0.9965	2.0 ppm	[5]
Photodetector	0.95	6.6 ppm	[49]
Chemiresistor	0.98	16 ppb	[50]
Chemiresistor	0.9901	0.42 ppm	[7]
Chemiresistor	0.993	3 ppm	[51]

## Data Availability

The original contributions presented in this study are included in the article. Further inquiries can be directed to the corresponding authors.
